# Editorial: What does experimental pharmacology and drug discovery look like in 2035?

**DOI:** 10.3389/fphar.2024.1504817

**Published:** 2024-11-29

**Authors:** Maria Serena Fabbrini, Riccardo Vago

**Affiliations:** ^1^ B&F BioConsulting, Monza, Italy; ^2^ Division of Experimental Oncology, Urological Research Institute (URI), IRCCS San Raffaele Scientific Institute, Milan, Italy; ^3^ Faculty of Medicine and Surgery, Università Vita-Salute San Raffaele, Milan, Italy

**Keywords:** experimental pharmacology, drug discovery, future therapeutic approaches, cutting-edge technologies, personalized drug development

**FIGURE 1:**
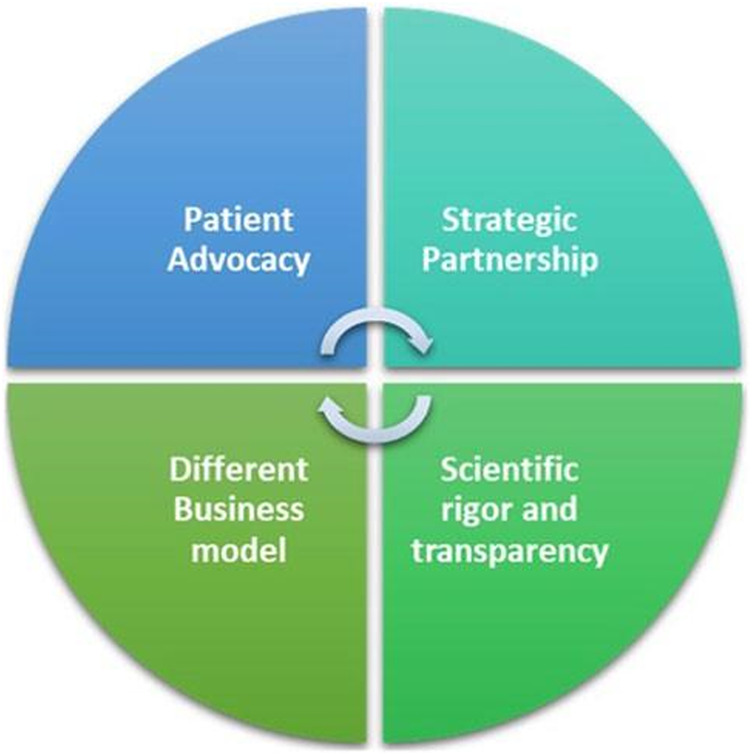
Fondazione Telethon’s key factors for a synergic workable alternative to the profit-driven pharmaceutical model.

While launching this Research Topic, Elevidys^®^ recombinant gene therapy to deliver a synthetic gene expressing a micro-dystrophin, as a single intravenous dose, was approved through the FDA Accelerated Approval pathway that leads to quicker approval of drugs for serious life-threatening diseases with high unmet need, as in Duchenne muscular dystrophy (Hoy, 2023). Telethon Foundation charity is a frontline institution in this area contributing here with a mini-review indicating the bottlenecks and possible solutions to make gene therapies available to rare disease patients thanks to the help and support of all the stakeholders including caregivers, parent’s advocacies, pharmaceutical companies, and regulatory agencies within an ecosystem starting from excellence in science (Vavassori et al.) (Figure 1). Co-funded by the European Union, JARDIN https://jardin-ern.eu - in coordination with other initiatives - could make rare diseases healthcare and research in the EU a model for the rest of the world**.** Together is better!

CRISPR-Cas9 technology is revolutionizing our therapeutic approaches and, by targeting the BCL11A erythroid-specific enhancer, allowed genetic editing of CD34^+^ hematopoietic stem and progenitor cells, thus reactivating the production of fetal hemoglobin (HbF) to fight beta-Thalassemia (Frangoul et al., 2021). The regulatory agencies FDA, EMA and the (UK) MHRA approved this treatment for transfusion-dependent patients or those affected by sickle cell disease with recurrent vaso-occlusive crises (Parums, 2024). Future appears to be already here.

Among risks of these treatments might be a wrong insertion of the exogenous recombinant gene, possibly beneath an oncogene, leading to tumorigenesis. DNA and RNA dual base editors’ tools can simultaneously introduce changes from C to T and A to G, deriving from a CRISPR modified system to allow a higher editing precision, as we learn from “The advance of base editing in genetic disorder treatment” by Xu et al. which will need to show further confirmatory studies *in vivo*.

Preventive medicine is increasingly gaining traction as a critical component of healthcare, emphasizing the importance of disease prevention in addition to treatment. This approach is well known in Traditional Chinese Medicine (TCM) and herbal medicine flavonoids possess numerous potential targets, but their mixed composition and absence of specificity/selectivity may hinder their broader use. Bioinformatics combined with computer-aided drug discovery and double-blind clinical studies should become an integral part of the development of small single therapeutic compounds in future TCM (Huang et al.).

Novel potential therapeutic treatments, mentioning the artificial intelligence-assisted, multidisciplinary approaches will need to integrate the wide multi(omics) data we are generating, to develop a precision medicine able to exploit the most advanced molecular/chemical tools.

In this context, the future of pharmacology leading to personalized medicine goes also through repositioning approved drugs’ use, to speed-up the whole process while drastically reducing costs. Tools such as “omics” and machine deep learning technology will drive our better understanding on drug mechanism(s) of action, thus facilitating the repurposing process. Acetylsalicylic acid, firstly commercialized against pain, as antipyretic and anti-inflammatory drug, then observed to be active in preventing cardiovascular diseases like myocardial infarctions or stroke, has a long versatile future ahead: lately aspirin has been proposed to play a role in reducing cancer incidence and metastatic spread (Montinari et al., 2019; Sikavi et al., 2024). Being an irreversible inhibitor of cyclooxygenase, aspirin blocks the prostaglandins (PG) and thromboxane A2 (TXA_2)_ synthesis, interfering with multiple pathways leading to disease onset. As reported by (Patrignani et al.) aspirin counteracts PGE2 and TXA_2_ biosynthesis in platelets and myofibroblasts both being enhancers of proliferative and migratory capabilities in the tumor microenvironment. Clinical trials follow-up is assessing the impact of low-dose aspirin in hereditary colon cancer CAPP3 known as Lynch syndrome (https://www.capp3.org/). Clopidogrel, one of the ADP receptor antagonist family, normally recommended for the reduction of atherosclerotic events, including myocardial infarction and ischemic stroke, which forms a disulfide bridge with extracellular cysteines of the P2Y_12_ receptor, could counteract with other P2Y_12_ inhibitors in patients with sporadic cancer. Drug repurposing will greatly impact new uses of “old” or investigational drugs that may result from accidental discoveries of off-targets such as with thalidomide, later repurposed to treat leprosy and multiple myeloma. Hundreds of drugs have moved into clinical trials during COVID-19 and four drugs received FDA emergency use authorization, with an additional 15 drugs being recommended for off-label use (Patrignani et al.). SARS-CoV2 treatment is the subject of computer-aided drug design (CADD), used in modelling drug-target interactions for inhibiting the main endo-protease M^pro^ (Dai et al.), which is a potential powerful explorative predictive tool, despite safety, preclinical data, pharmacodynamics, and clinical studies need to be conducted to identify the lead drug candidate. Another *in silico* approach was used for designing and then synthesizing tyrosinase inhibitors which underwent high-resolution mass spectroscopy and Fourier infrared analyses (Ahmad et al.). The purified compounds were refluxed with aldehydes and ketones in a step-by-step innovative and cost-effective process to identify a lead compound. Mammalian tyrosinase is a single transmembrane-spanning polypeptide and in humans, tyrosinase is sorted to melanosomes where it is involved in the melanin synthesis and is a biomarker for several skin diseases, as an anti-melanogenesis target.

French et al. developed a live-cell assay, termed “ClickArr”, that simultaneously reports recruitment of both β-arrestin 1 and 2 isoforms, as they compete for interaction with delta opioid receptor (δOR). This drug screening platform provides researchers tools to design drugs that may optimize analgesic efficacy while reducing tolerance development. Beta-arrestin-1 and 2 indeed, are multifunctional intracellular isoforms that modulate cell signaling by regulating desensitization, internalization, and re-sensitization of G protein-coupled receptors (GPCRs), including δORs. Their interactions with δORs are critical to understand receptors’ main function in a variety of pathological processes, such as migraine, neuropathic pain, and alcohol abuse disorder. Different δOR agonists preferentially recruit specific β-arrestin isoforms and this specific recruitment plays a crucial role in determining the functional outcomes of receptor activation to trigger subsequent signaling pathways (Pradhan et al., 2016). The nuanced roles of β-arrestins in δOR signaling gives the opportunity for developing “biased” agonists which may selectively activate beneficial signaling pathways while minimizing adverse effects. Furthermore, this modelling could be developed for other GPCRs beyond the δOR, extending it to a diverse array of prospective drug targets.

The next 10 years of experimental pharmacology will be marked by significant challenges that require innovative solutions to be overcome. Integration of new technologies and collaborative efforts across disciplines can offers promising directions for improving the drug discovery process.
